# Various Aspects Involved in the Study of Tooth Bleaching Procedure: A Questionnaire–Based Study

**DOI:** 10.3390/ijerph19073977

**Published:** 2022-03-27

**Authors:** Amalia Mazilu Moldovan, Violeta Popescu, Corina Violeta Ionescu, Stanca Cuc, Antarinia Craciun, Marioara Moldovan, Diana Dudea, Anca Stefania Mesaros

**Affiliations:** 1 Physics and Chemistry Department, Technical University of Cluj-Napoca, 400641 Cluj-Napoca, Romania; amalia.mazilu@gmail.com (A.M.M.); violeta.popescu@chem.utcluj.ro (V.P.); 2 Department of Conservative Odontology, Iuliu Hatieganu University of Medicine and Pharmacy, 400001 Cluj-Napoca, Romania; 3 Raluca Ripan Chemistry Research Institute, Babeș Bolyai University, 400294 Cluj-Napoca, Romania; stanca.cuc@ubbcluj.ro (S.C.); marioara.moldovan@ubbcluj.ro (M.M.); 4 Department of Prosthetic Dentistry and Dental Materials, Iuliu Hatieganu University of Medicine and Pharmacy, 400006 Cluj-Napoca, Romania; antarinia@yahoo.com (A.C.); ddudea@umfcluj.ro (D.D.); ancames@yahoo.com (A.S.M.)

**Keywords:** questionnaire, tooth bleaching, patients, practitioners, tooth discoloration, satisfaction

## Abstract

A beautiful smile is an important feature when it comes to a pleasant appearance of the face, and one of the most common situations that drive patients to book a dental appointment is tooth discoloration. Tooth bleaching is the treatment of choice for extrinsic tooth discoloration, as it is a cheap, fast, and minimally invasive procedure. This study aimed to provide comparative information on the perceptions of both patients and dentists regarding different whitening methods and on the factors involved in people’s willingness to recommend and use a bleaching procedure. In addition to this, this study evaluated the degree of satisfaction in relation to the bleaching methods and materials used; it also evaluated the following: negative side effects, economic characteristics and the patients’ perceptions of the aesthetic appearance of their dental arches, especially tooth color. The subjects that participated in the present study were selected based on their background and were then divided into two categories. The first group consisted of 120 patients who had received tooth-bleaching treatments in dental clinics during the study and the second group consisted of 127 dentists. A conventional sampling method was used. The study aimed to define a relationship between multiple aspects of the tooth-bleaching procedure, including the patients’ desires and their general knowledge of this procedure. Tooth color and the way it changes is a very important factor that motivates patients to come to the dentist for whitening procedures. Patients showed the highest levels of satisfaction with the results of in-office bleaching procedures. In the group consisting of dentists, satisfaction levels were higher for the procedure of home bleaching supervised by a dentist. Factors influencing the choice of bleaching materials are appreciated differently by dentists and patients. Furthermore, the rate of patients using OTC (over the counter) products was found to be high. Further research is needed to find more effective and safer alternatives to home tooth-bleaching procedures.

## 1. Introduction

A bright and white smile is an important feature of a person’s face and has very important implications. It is associated with self-image, social interactions and even the mental health of patients [1,2,3]. Due to globalization and rapid modernization in all areas, both men and women have become very aware of their appearance and, therefore, attach a great deal of importance to every detail that will improve their looks [4,5,6,7]. Assessing the aesthetic aspects in order to establish a treatment plan is one of the priorities of the Medical Act in dentistry.

Therefore, in dental medicine, numerous methods of communication have been introduced to allow the dentist to analyze the psychological and informational features of the patient. Patients can then perform a more in-depth analysis of their teeth and their facial appearance, to understand all the treatment options offered by the specialist and their limitations, and even see a preview of the treatment’s results (either in a virtual or physical format) [8,9].

Questionnaires play an important role in doctor–patient communications regarding the patient’s perception of their dental arches. Aesthetic analysis questions may be included in the more extensive questionnaires or may take the form of cosmetic questionnaires addressed to patients in the office.

In research conducted by Khan et al., a comparative assessment was performed of the perception and interpretation of changes in the shape, color and positioning of teeth; facial symmetry or dental arches; tooth malpositioning; and occlusal abnormalities [10]. This research was conducted in the form of questionnaires addressed to people with different levels of knowledge in the field of dentistry (e.g., untrained people in this field of activity vs. practitioners). The results of this research showed that dentistry practitioners, when assessing deviations, often observe them in a more critical manner than patients when the deviations are compared to normal appearance. The conclusions were very varied when it came to the timeliness of the therapy and the preferred treatment methods, because of the experience of the dentistry practitioners and their specialized information base.

When the appearance of the teeth is negatively affected by various dental conditions, this can result in a loss of self-esteem and the deterioration of both physical and mental health [1,11,12,13].

One of the most common situations that drive patients to come to the dental office is tooth discoloration, which can take on different intensities and etiologies and can include entire dental arches (generalized tooth discoloration) or be confined to one or more teeth (localized tooth discoloration). There are many treatment options for changes in tooth color, including tooth-bleaching procedures. Tooth bleaching is currently the treatment of choice for extrinsic tooth staining, as it is a cheap, fast, and minimally invasive procedure [14,15].

Tooth bleaching can be performed by practitioners in the dental office or by using over-the-counter products purchased by the patient and used at home (toothpaste, rinsing solutions, strips containing whitening agents, chewing gum, etc.). The most widely used method of tooth bleaching is therapy initiated, recommended, and supervised by a doctor, followed by continued treatment by the patient at home [16,17].

This study aimed to obtain comparative information on the perception of both dentists and patients on the following issues related to tooth-bleaching procedures:

- Different whitening methods, recommended mainly by dentists and chosen by patients.
- Factors involved in recommending and using tooth-bleaching methods and bleaching materials.
- Satisfaction with the bleaching methods and materials used.
- Patients’ self-perception of the aesthetic appearance of their dental arches, especially regarding tooth color.

This study was complex and addressed both patients and dentists, and the opinion poll was the most important aspect of this piece of social research.

We started from the null hypothesis that there would be no statistical differences between the answers given by dentists and patients.

## 2. Materials and Methods

### 2.1. Study Design

Two web-based surveys were designed and distributed via Microsoft Forms. One contained 20 questions and was addressed to dentists and the second one contained 24 questions and was addressed to dental patients. They contained questions designed to address the six aforementioned research directions. 

In the two questionnaires, the questions were formulated specifically for each group (doctors/patients) and took into account both the different stages of assessing the procedures and the different levels of understanding of the terminology. The structure of the questions was either of a dichotomous type (i.e., yes/no answers) or an evaluation, using a scale from 1 to 5. Study participants were invited to enroll in the study starting in March 2020 [18] (Supplementary Materials Files S1 and S2), and the questionnaires were opened to be answered for a period of 6 months.

The questionnaires were designed with reference to the questionnaire on the quality of life related to oral health (McGrath and Bedi) [19], which contains 19 questions based on an awareness of dental aesthetics [2]. In our study, questions were asked about the physical, functional, social, psychological and knowledge aspects of the participants [20]. Prior to distribution, the validity and content of the questionnaire were assessed. The questionnaire was pilot tested on 10 dentists and 10 patients, and the variability was found to be 0.7 (fair agreement).

The questionnaire was then distributed via the internet (e-mail or social media) and on different dentistry forums and discussion rooms. Dentists were also asked to forward a link to the patient-oriented questionnaire to patients or to post the link in their office waiting rooms. Participants were voluntarily involved in this study, and informed consent was obtained from each of them. Prior to completing the questionnaire, each participant was determined to be able to read and, in the case of acceptance, sign a form containing a brief description of the purpose of the study and the rights and informed consent of each participant. All participants (dentists and patients) were informed that no personal data would be collected and that the survey would be completed anonymously to comply with the GDPR regulations. The survey did not ask for the names of practices, practitioners or patients and it was distributed in 3 languages (Romanian, English, and French) in order to reach a larger demographic.

The first group consisted of 120 patients, aged between 18 and 57 years old, who were receiving consultation and/or treatment in dental clinics during this study. The second group consisted of 127 dentists, endodontists and general dentistry practitioners aged between 25 and 54 years old. In total, the study was based on 247 subjects who answered the questionnaire in full.

We received approval for this study from the Ethics Commission at the University of Medicine and Pharmacy, Cluj-Napoca, under the approval number 134/11 May 2021. As no experiments were carried out on participants and our study followed GDPR regulations and the Helsinki Declaration, the Ethical Committee’s approval was obtained for publication purposes only.

We would like to note that no minor (under the age of 18) participated in this study. This was a criterion of exclusion from the study, since the tooth-bleaching procedure is not indicated for patients under this age and because the age of 18 is required for them to be legally responsible and give their consent.

### 2.2. Statistical Analysis

Answers were stored in a Microsoft Excel database, created after pairing the correspondent questions from the two databases formed after the surveys. Members of the research group reviewed the data for accuracy. Descriptive statistics were calculated, such as frequencies, means, medians, and standard deviations. A one-way repeated measures ANOVA test was used to test the differences between the investigated groups. The chi-square (Bartlett Test) and Brown–Forsythe exact tests were used; they use the group median instead of the group mean to compare the distributions of the category-inflicted variables. Student’s *t*-tests were used for post-hoc comparisons. The statistical package used for data analysis was Prism version 4.00 for Windows, GraphPad Software, San Diego, CA, USA.

## 3. Results

Of the 120 patients who answered the questionnaire, 16 were French, 18 were Romanian and 86 were English; whilst for the practitioners’ questionnaire, out of a total of 127, 32 were Romanian and 95 were English. Table 1 summarizes the characteristics of the participants in this study.

The one-way ANOVA test showed statistically significant correlations between all parameters questioned among dentists who participated in the study (*p* < 0.0001), (n = 127, *p* = 0.0469). The variables investigated included the following: the practitioner’s age, use and preferences for various specific materials, recommendations in various clinical scenarios, indications, side effects, patient satisfaction, whitening efficacy, cost, and the number of sessions required.

In the *t*-test, when calculating the correlation between age parameters and the use of tooth-whitening materials among the practitioners who responded to the survey, a statistically significant difference was observed (*p* = 2.02993 × 10^−15^); 72% of the participating dentists had indicated and performed dental treatments over the last two years, and most of them were aged between 25 and 30 years, and have had less than 5 years of practical experience.

When analyzing the parameters related to the dentists’ choices in selecting the tooth-bleaching method indicated for their patients, the one-way repeated measures ANOVA test showed a statistically significant difference, both in the group of parameters (*p* < 0.0001) and in the group of dentists (*p* < 0.0001); consequently, in terms of the timeliness of the therapy and the preferred treatment methods, the results were very varied, given the specialized information base and the experience of the dental practitioners.

**Figure 1:**
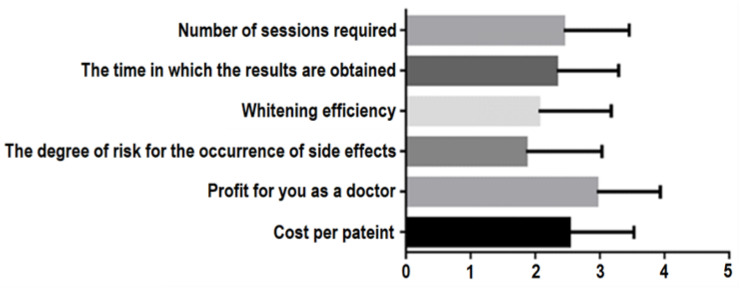
Statistical analysis of in-office bleaching method parameters in relation to the listed criteria (where 1 is the most important and 4 is the least important of the criteria).

The least important criterion found to be appreciated by the dental practitioners was profit, followed by the cost per patient, the number of sessions required, the time in which results were obtained and the whitening efficiency. The most important criterion was the degree of risk associated with the side effects (Figure 1).

**Figure 2:**
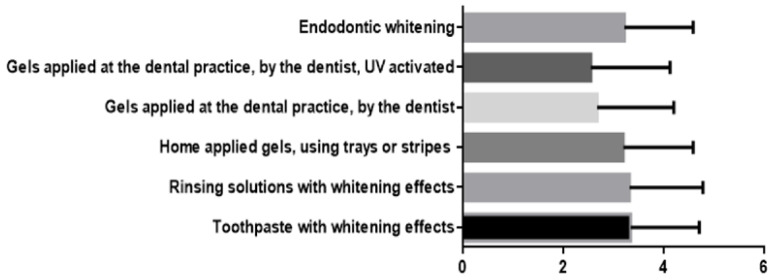
Statistical analysis of practitioners’ choice of the tooth-bleaching procedure (where: 1—most common; 5—least common).

In the analysis of the parameters related to the order in which doctors prefer to indicate the use of tooth-bleaching procedures, there was a statistically significant difference (*p* < 0.0001), as shown in Figure 2. Toothpastes with whitening effects were the least indicated procedure, followed by rinsing with bleaching-effect mouthwashes; internal bleaching of endodontic-treated teeth; whitening gels, applied by the patient at home, using individual trays and high carbamide peroxide concentration bleaching gels, applied by the doctor in the dental office. The bleaching treatment using light-activated whitening gels was the most common procedure that the practitioners chose for tooth bleaching.

**Figure 3:**
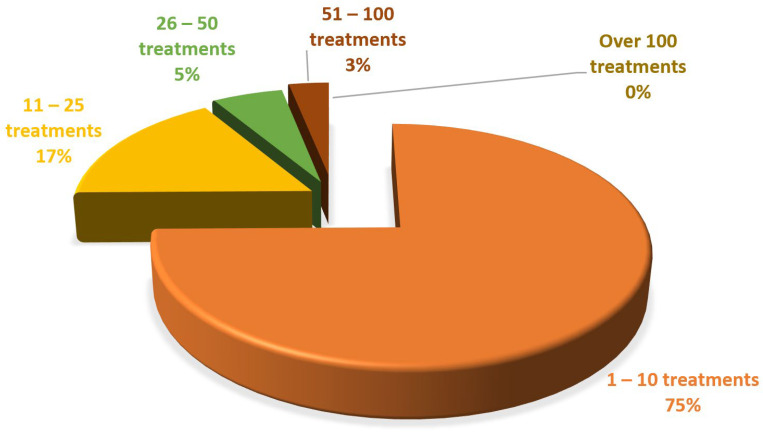
Number of bleaching procedures performed by doctors in their last 2 years of practice.

The analysis of the frequency of bleaching procedures performed by dentists in dental offices showed that most practitioners had performed up to 10 whitening treatments in the last 2 years of practice, as shown in Figure 3.

There is a statistically significant difference between the rate of whitening treatments performed at the express request of patients and the percentage of all bleaching treatments indicated by dentists (*t*-test, *p* = 9.1552 × 10^−09^). This indicates that the differences between the two variables are correlated.

**Figure 4:**
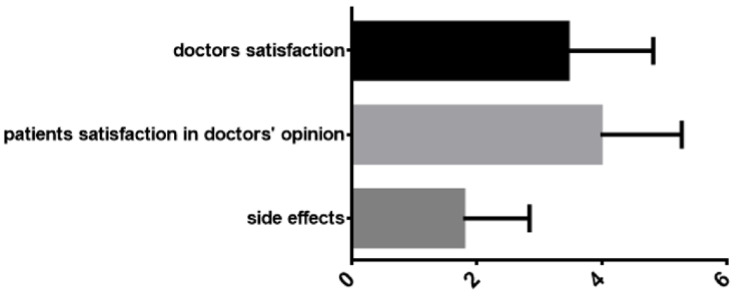
Satisfaction of dentists in relation to the degree of satisfaction of patients after whitening treatment (in the opinion of the dentists) and the rate of post-whitening side effects (where 1 = less important, 4 = most important).

The degree of dentist satisfaction with whitening treatments was in close statistical correlation with the degree of patient satisfaction with dentists and with the extent to which patients experienced side effects from bleaching procedures, both in the parameter group (*p* < 0.0001) and in the group of patients (*p* < 0.0001), as Figure 4 shows.

There is a statistically significant difference (*p* = 0.0014) between the number of patients (n = 120) included in the study and the parameters discussed (*p* < 0.0001). This highlights a strong correlation between the following: the patients’ ages; their aesthetic concerns; their use of either home-whitening treatments or dental practice treatments; the types of products used; the price of products and occupational treatments; the outcome and options of tooth-whitening treatments used and the decisions made in various scenarios (e.g., the presence of different types of dental restorations or orthodontic appliances).

**Figure 5:**
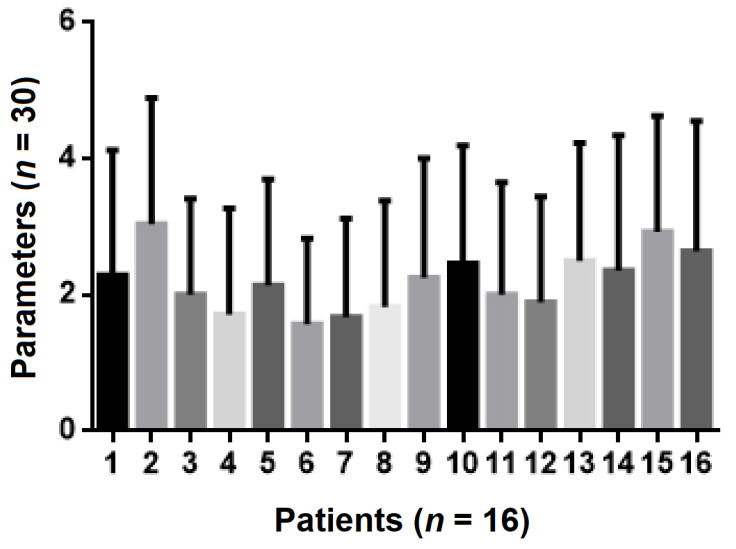
Anova repeated measures parameters analysis in patients who underwent home tooth whitening and office bleaching. Correlation between the number of patients who underwent home tooth whitening and those who underwent in-office bleaching.

Within the group of patients (n = 16) who underwent tooth bleaching, both at home and in the dentist’s office, a statistically significant correlation was found (*p* = 0.0093) (Figure 5). Additionally, all parameters (age of patients, aesthetic demands, results, options of whitening treatments, and the cost of home-bleaching procedures) included in the analysis showed statistically significant differences (*p* < 0.0001). The exception to this was patients who had undergone in-office bleaching and their views on specific crown restorations or on the impact of orthodontic treatment on tooth- whitening decisions. These results are predictable, as most patients are not trained to have objective opinions about different treatment options, unless they are given professional counsel. Emphasis should also be placed on the amount of money that patients spend on bleaching procedures.

**Figure 6:**
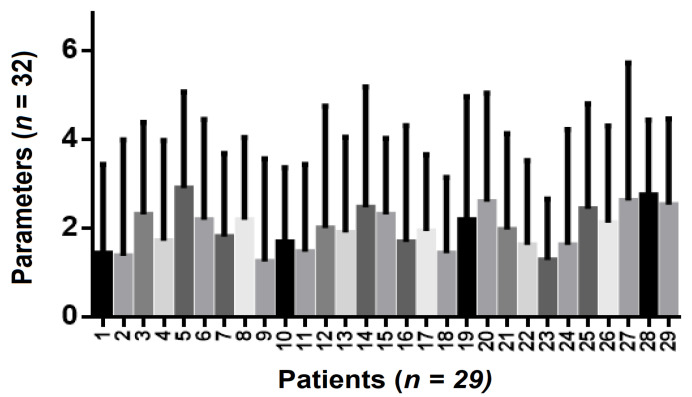
Anova repeated measures parameters analysis in patients with “in the office bleaching” Correlation between the number of patients who underwent in-office bleaching compared and the parameters studied.

In the group of patients (n = 29) who received in-office bleaching procedures, there is a statistical difference between their number in relation to the parameters included in the study (*p* = 0.0023). There are also statistically significant differences between all the parameters included in the study (*p* < 0.0001) (Figure 6).

**Figure 7:**
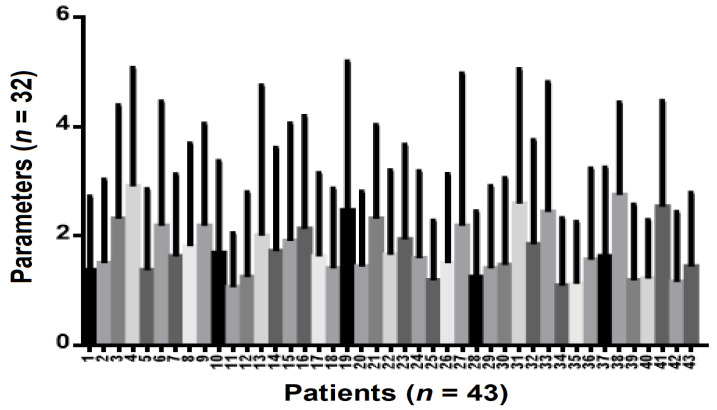
Anova repeated measures parameters analysis in patients with home tooth bleaching. Correlation between the group of patients who underwent home-tooth bleaching and the parameters studied.

In the group of patients with home-bleaching treatments, the one-way ANOVA test revealed a statistically significant correlation (*p* = 0.005) for all the parameters investigated in this study (*p* < 0.0001) (Figure 7).

**Figure 8:**
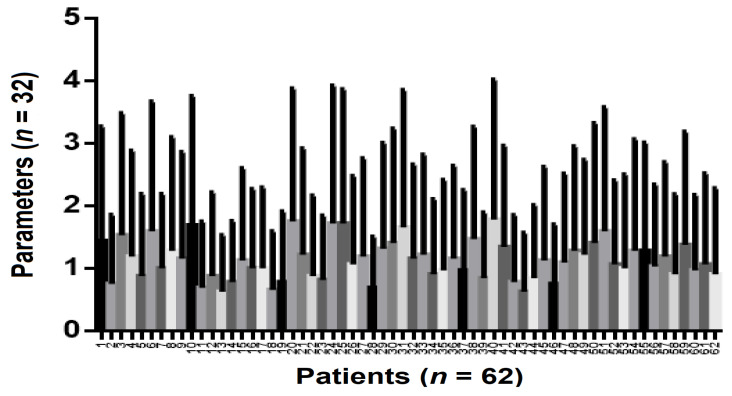
Anova repeated measures parameters analysis in patients without any bleaching treatment. Correlation between the group of patients without any bleaching treatment and the studied parameters.

In the group of patients who did not undergo any bleaching, there is a statistically significant difference compared to the other groups of patients analyzed (*p* = 0.0005). At the same time, there are statistically significant differences in relation to the whole group of patients interviewed in this study (*p* < 0.0001), as shown in Figure 8.

The degree of satisfaction is more significant in the group of patients who underwent tooth-bleaching procedures in the office (n = 29, average satisfaction degree = 4.034482759) than in the group of patients who underwent tooth-bleaching procedures at home (n = 43, degree of satisfaction-environment = 3.302325581).

**Figure 9:**
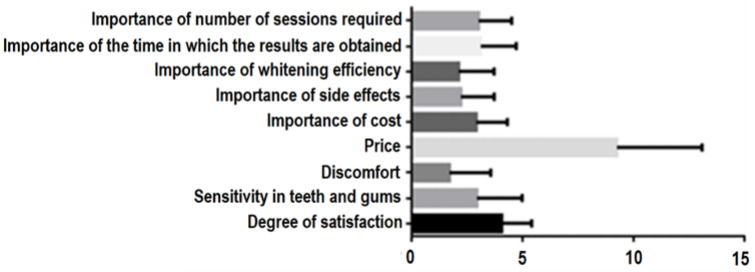
Correlation between the degree of satisfaction of in-office tooth bleaching and the parameters studied.

There were also significant statistical correlations between the satisfaction of pa-tients who were treated in the office (*p* < 0.0001) in relation to the following: the importance assigned by patients to this treatment, the efficiency of the whitening procedures, the side effects, the number of sessions necessary, the time in which the results were obtained, the cost, the discomfort, the sensitivity of teeth and gums, and the amount of money spent. The Brown–Forsythe test (*p* < 0.0001) and the Bartlett test (*p* < 0.0001) both show equal variations within the groups of parameters analyzed (Figure 9).

**Figure 10:**
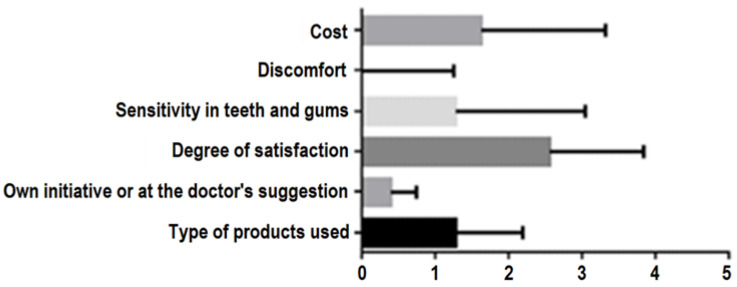
Correlation between the satisfaction of home-tooth bleaching and the studied parameters.

Similar results were obtained when investigating the correlation between the patients’ satisfaction with home-bleaching treatments and the type of products used, the patients’ decisions, the results related to sensitivity, discomfort, and the price (*p* < 0.0001). The Brown–Forsythe (*p* < 0.0001) and Bartlett (*p* < 0.0001) tests show that the differences between the calculated averages are statistically significant (Figure 10).

**Figure 11:**
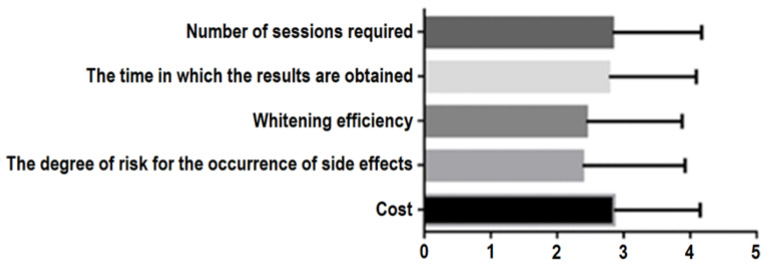
Patients’ opinions in relation to the chosen bleaching treatment.

There is a statistically significant difference both in the analysis of patients’ options in relation to their choice of whitening procedure (*p* = 0.0017, one-way repeated measures ANOVA Test) and in the correlation of patient options relative to the whole group of patients studied (*p* < 0.0001, one-way ANOVA test). The most important factor in choosing which bleaching procedure should be indicated was the cost of the treatment, followed by the number of sessions required, the time in which the results were obtained, the whitening efficiency, and the degree of risk associated with the side effects. An interesting observation was made regarding the most important predictable factors that were found in this study in relation to the last two positions (Figure 11).

A strong statistical correlation (*p* = 2.41769 × 10^−12^) was observed between the age of the patients and their satisfaction with their teeth after having undergone treatment (the young patients were more satisfied).

**Figure 12:**
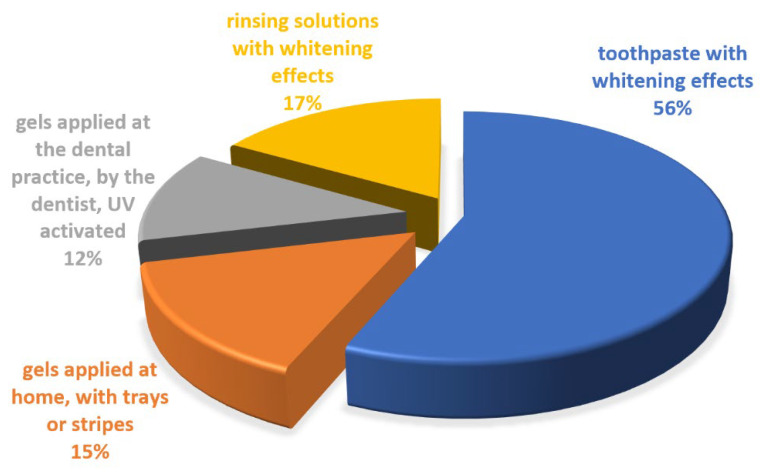
Methods of tooth bleaching chosen by patients.

Comparing the bleaching procedure to the frequency of use by patients, the toothpaste with whitening effects occupied the first position, followed by rinsing solutions with whitening effects; home-bleaching procedures, using custom trays and in-office whitening using light-activated or laser-activated bleaching gel (as shown in Figure 12).

## 4. Discussion

This study was conducted to define the relationship between multiple aspects of tooth-whitening procedures, including patients’ desires and general knowledge about these procedures. It should be noted that the concerns of patients about the aesthetic appearance of their teeth occupied an important place, as did the cost, duration, and the undesirable side effects of whitening treatments. The null hypothesis of this study was rejected: there are statistical differences between the answers given by dentists and patients.

Out of the patients who participated in this study, the ones who had experience in whitening treatments, mostly preferred the tooth-whitening procedures conducted at home, followed by subjects who underwent tooth whitening in the office. Similar results were also investigated and disclosed by Alotaibi et al. [21] and Aldakheel et al. in 2018 [22]. Another study by Ahmed et al. assessed the attitudes of patients towards in-office bleaching and whitening procedures with OTC products, as well as the awareness of patients about the indications and side effects of over-the-counter tooth whitening products among the inhabitants of Riyadh, KSA [23].

The results of our study strongly indicate that dentists’ awareness and observational capabilities are far greater and a lot more critical than those of patients in assessing deviations from normal appearance. Regarding the timeliness of the therapy and the preferred treatment methods, the results were very varied, given the specialized information base and the experience of the professionals. Tooth sensitivity is one of the most common side effects of tooth-whitening procedures due to the action of oxygen free radicals resulting from the decomposition of the carbamide peroxide. Some studies have explained this by citing the ability of peroxide to diffuse through the dentinal tubules to the pulp chamber [24,25,26,27,28].

Another adverse effect of tooth-whitening procedures is the morphological changes of the enamel, caused by the loss of calcium ions in its structure [29,30]. Gonzalez-Lopez et al. reported that tooth whitening altered the organic composition of the enamel and decreased the content of phosphates and carbonates, thus making the enamel surface rough and uneven [31,32].

Knowing and understanding the chemical mechanisms of the tooth-bleaching procedures is very important for dentists. Knowledge of the physiological process involved in the selection of tooth shade will allow practitioners to better communicate with the technical staff and to consider their patients’ opinions in the process of choosing tooth color before cosmetic correction. When recommending or prescribing oral care products that are known to cause staining, it is important to warn patients of the potential side effects.

Of the 127 doctors surveyed, 72% have indicated and performed whitening treatments so far in the last two years, out of which 75% of them have conducted between 1 and 10 treatments. These percentages indicate dentists’ knowledge of whitening methods and their current use in their own practices. Moreover, many of these are performed at the request of patients. Out of the 120 patients surveyed, 37% used tooth- whitening methods. Eighty-six percent of them tried the technique on their own initiative, and only fourteen percent tried it at the suggestion of the dentist. The most common treatments were found to be pastes or rinsing solutions (39%), natural remedies (18%), and whitening strips (43%).

Of the patients surveyed, 24% benefited from whitening treatments in the office. In conclusion, patients use commercially available tooth-whitening methods, often without a clear recommendation from the dentist. This can have unfavorable consequences, as patients are not always able to appreciate the etiology of a dyschromia and, subsequently, the most appropriate treatment.

Further research has concluded that the most common treatment chosen by dental practitioners is a tooth-whitening procedure conducted in the office, using bleaching gels with a concentration of 10–20% CP (carbamide peroxide) and 37% HP (hydrogen peroxide) for both vital and non-vital techniques [12,33].

Due to technological advances, a variety of whitening procedures are now available for patients who want to improve the appearance of their teeth. Advances in aesthetic dentistry have given patients more options in order to obtain the best results. Despite many choices, we must keep in mind that people have different needs, as opposed to attitudes towards treatment [34,35,36,37,38].

Through their study, Siddiqui et al. concluded that many people like the idea of simply using natural bleaching products, even if it means using them in larger quantities or waiting longer in order to see results. In addition to this, they may believe that these products are convenient and inexpensive. Almost half of the respondents used one or more types of the traditional tooth-whitening methods, despite advances in aesthetic dentistry [39,40].

Because the variety of OTC products with a whitening effect is quite extensive, and because of their net advantages in terms of cost (which is much lower than professional products) and their relatively easy application, patients often prefer to use them, even though the results are often unsatisfactory.

The attention of researchers has increasingly focused on the study of the actions of natural plant extracts, which have been used since ancient times in both traditional Eastern and Western medicine for their anti-inflammatory and anti-bacterial effects [24,41,42,43,44,45].

Depending on the particularities of the questions and their addressability, the results can be used to establish treatment plans. Additionally, these results can be useful for an overall analysis of the perception of certain categories of deviations from aesthetic norms, for establishing trends in treatment requests, and for correcting them.

In correlating the results obtained from the two questionnaires, it was deemed necessary for future studies to include a question to investigate whether or not patients had reported any side effects to their dentist. Other points that have been suggested for consideration in future studies are to investigate to what extent doctors are interested in the possible occurrence of post-treatment side effects and to investigate what the most frequently adopted therapeutic attitude is. We also recommend investigating a larger number of respondents to significantly increase the accuracy of interpretations.

## 5. Conclusions

1. Opinion polls on tooth color and changes are important aspects that motivate socio-human research.

2. Patients’ satisfaction with the results of in-office bleaching procedures was higher compared with the results obtained after the home-whitening treatment with OTC (over the counter) products, although this procedure has produced fewer side effects such as tooth or gingival sensitivity. In the group of dentists, satisfaction was higher with home bleaching supervised by the dentist.

3. Factors influencing the choice of bleaching materials are appreciated differently by dentists and patients: dentists were reluctant to indicate these treatments in the presence of crown restorations and orthodontic appliances.

4. The rate of patients using OTC (over the counter) products was high. Given the limited number of participating patients, the correlation of these treatments with the etiology of tooth discoloration, and with the proper dosage of whitening products, their side effects should be limited. Further research is needed to find alternatives to home treatments that are more effective but do not inflict undesirable side effects.

## Figures and Tables

**Table 1:** Characteristics of the questionnaire participants: practitioners and patients.

	Practitioners	Patients
**Origin:**	O (%)	O (%)
Romanian	32	18
English	95	16
French	0	86
**Age:**	Years (SD)	Years (SD)
Mean	29 (8.6)	25 (8.4)
Median	31	29
Range	18–55	18–55

## Data Availability

Not applicable.

## Supplementary Materials

The following supporting information can be downloaded at: https://www.mdpi.com/article/10.3390/ijerph19073977/s1, File S1: Questionnaire for doctors; File S2: Questionnaire for patients.
