# Supplementary material for: Various Aspects Involved in the Study of Tooth Bleaching Procedure: A Questionnaire–Based Study
Source: Int J Environ Res Public Health. 2022 Mar 27;19(7):3977. doi: 10.3390/ijerph19073977 (PMC8997818; doi:10.3390/ijerph19073977)
Supplement: Supplementary file 1 [file ijerph-19-03977-s001.zip › File S1. Questionnaire for doctors.pdf]

QUESTIONNAIRE FOR DOCTORS

Your age:
Between 25-30
30-35
35-40
40-45
45-50
Over 50

1. Do you use dental whitening materials at your dental practice?
Yes
No

2. Do you indicate dental whitening treatments for your patients?
Yes
No
Sometimes

3. Choose the order in which you prefer to indicate the teeth whitening methods, using numbers from 1 to 5: 1. Most common - 5 Least common

- toothpaste with whitening effects ....
- rinsing solutions with whitening effects ....
- home applied gels, using trays or stripes ....
- gels applied at the dental practice, by the dentist......................
- gels applied at the dental practice, by the dentist, UV activated ....
- endodontic whitening ....

4. Among the following criteria, which is the order of importance you attach to the choice of whitening option indicated to patients. Write down numbers from 1 to 4, 1 most important, 4 least important of the criteria

- cost per patient ....
- profit for you as a doctor...............
- the degree of risk for the occurrence of side effects................
- whitening efficiency................
- the time in which the results are obtained...............
- number of sessions required..................

5. Do you consider that the presence of dental fillings influences your decision to recommend a whitening treatment?

YES/ NO

6. Do you consider that the presence of older prosthetic works influences your decision to recommend a whitening treatment?

YES/ NO

7. Do you consider that the presence of new prosthetic works influences your decision to recommend a whitening treatment?

YES/ NO

8. Have you indicated and performed dental whitening treatments for your patients?

YES/ NO

9. In patients with fixed orthodontic device do you recommend whitening treatment during the orthodontic treatment?

YES/NO

10. How many dental whitening treatments have you indicated and performed for your patients in the last 2 years?

1-10 treatments
10-25 treatments
25-50 treatments
50-100 treatments
Over 100 treatments

11. What would be the percentage of endodontic whitening treatments out of all the whitening treatments you have indicated and performed?

Under 10%
Between 10 and 25%
Between 25 and 50 %
Between 50 and 75 %
Over 75%

12. What would be the percentage of dental whitenings performed at the patient's express request?

Under 10%
Between 10 and 25%
Between 25 and 50 %
Between 50 and 75 %
Over 75%

13. To what extent were you satisfied with the results obtained from the whitening treatments?

Under 10%
Between 10 and 25%
Between 25 and 50 %
Between 50 and 75 %
Over 75%

14. To what extent have your patients been satisfied with the results obtained from the whitening treatments?

Under 10%
Between 10 and 25%
Between 25 and 50 %
Between 50 and 75 %
Over 75%

15. To what extent have your patients suffered side effects from dental whitening treatments?

Under 10%
Between 10 and 25%
Between 25 and 50 %
Between 50 and 75 %
Over 75%
