# Supplementary material for: Various Aspects Involved in the Study of Tooth Bleaching Procedure: A Questionnaire–Based Study
Source: Int J Environ Res Public Health. 2022 Mar 27;19(7):3977. doi: 10.3390/ijerph19073977 (PMC8997818; doi:10.3390/ijerph19073977)
Supplement: Supplementary file 1 [file ijerph-19-03977-s001.zip › File S2. Questionnaire for patients.pdf]

QUESTIONNAIRE FOR PATIENTS

1. Are you unhappy with the appearance of your smile?

YES/ NO

2. Among the following criteria, which is the importance that you attach to the aesthetics of your smile. Write down with numbers from 1 to 5, 1 most important, 5 least important of the criteria

- Size / shape of the teeth..............................
- Dental alignment..
- Unaesthetic dental treatments............................
- Teeth color..
- Gum appearance..

3. To what extent are you satisfied with the color of your teeth

1- Highly dissatisfied

2- Dissatisfied

3- Undecided

4- Satisfied

5- Highly satisfied

4. Have you ever used teeth whitening at home?

YES/ NO

If so, what are these?

Toothpaste, whitening rinsing solution

Natural remedies (lemon, bicarbonate)

Whitening stripes

5. Has whitening at home been done on your own initiative or at the doctor's suggestion?

Own initiative

Doctor's suggestion

6. The degree of satisfaction on a scale from 1-5 of the results obtained after the whitening treatment at home.

1 very unsatisfied - 5 very satisfied

7. Did you have a high teeth and gums sensitivity after the whitening procedure at home?

YES/ NO

8. On a scale from 1 to 5 what was the degree of satisfaction with the discomfort created by this sensitivity?

1- Minimum

2- Low

3- Fair

4- High

5- Maximum

9. What was the amount you invested in home whitening treatment?

Under 20 EUR

Between 20 and 39 EUR

Between 40 and 59 EUR

Between 60 and 79 EUR

Between 80 and 100 EUR

Over 100 EUR

10. Have you ever had a teeth whitening procedure at your dental practice?

YES/NO

11. The degree of satisfaction on a scale from 1-5 of the results obtained after the whitening treatment at the dental practice.

1 very unsatisfied - 5 very satisfied

12. Did you subsequently had a high teeth and gums sensitivity after the whitening procedure at the dental practice?

YES/ NO

13. On a scale from 1 to 5 how high was the discomfort created by this sensitivity?

1- Minimum

2- Low

3- Fair

4- High

5- Maximum

14. What was the price of the whitening treatment at the dental practice?

Endodontic whitening / tooth / session

10-19 EUR

20-29 EUR

30-39 EUR

Over 40 EUR

Dental practice treatment / session

100-124 EUR

125-144 EUR

145-164 EUR

165-199 EUR

Over 200 EUR

Dental practice treatment with UV activated gels/ laser/ session

165-184 EUR

185-199 EUR

200-224 EUR

225-264 EUR

265-299 EUR

Over 300 EUR

15. To what extent (on a scale from 1 to 5) did you attach importance to the following factors when choosing the whitening option used (1 very important - 5 unimportant)

- cost
- the possibility of side effects
- whitening efficiency
- the time in which the results are obtained
- number of sessions required

16. Do you consider that the presence of dental fillings can influence the decision of having a whitening treatment?

YES/NO

17. Do you consider that the presence of dental work can influence the decision of having a whitening treatment?

YES/NO

18. In patients with fixed orthodontic devices (brackets) do you consider that whitening treatments can be performed during the wearing of the device?

YES/NO

19. Among the whitening options, you have used so far:

- toothpaste with whitening effects
- rinsing solutions with whitening effects
- gels applied at home, with trays or stripes
- gels applied at the dental practice, by the dentist, UV activated
